# Targeting CXCR1/2 suppresses T_H_2/T_H_17 cell responses and inhibits dual-pathology allergic lung inflammation

**DOI:** 10.1016/j.jacig.2026.100726

**Published:** 2026-04-30

**Authors:** Koa Hosoki, Annamalai Govindhan, John M. Knight, Sanjiv Sur

**Affiliations:** a Department of Medicine, Immunology, Allergy and Rheumatology, Baylor College of Medicine, Houston, Tex; b Department of Pathology and Immunology, Biology of Inflammation Center, Baylor College of Medicine, Houston, Tex

**Keywords:** Allergic inflammation, CXCR, proliferation, T_H_2 cells, T_H_17 cells

## Abstract

**Background:**

Many chronic inflammatory diseases are driven by T_H_2 and T_H_17 immune responses and represent a major public health burden. While monoclonal antibodies against individual T_H_2 or T_H_17 cytokines or receptors show clinical benefit, their efficacy is limited by overlapping T_H_2/T_H_17 pathology. This highlights the need to identify shared upstream pathways whose inhibition could suppress both responses simultaneously.

**Objective:**

We sought to elucidate the role of CXCR1/2 in recruitment and proliferation of T_H_2 and T_H_17 cells during allergic airway inflammation.

**Methods:**

Studies used mice sensitized and challenged with cat dander extract, inducing dual T_H_2/T_H_17 lung inflammation. Expression of T_H_2, T_H_17, CXCL, and CXCR was quantified, and *ex vivo* CXCL stimulation of lung single-cell suspensions was used to assess T_H_2 and T_H_17 proliferation.

**Results:**

Allergen challenge upregulated *Cxcl1/2/3/5* mRNA, and increased CXCR1/2^+^ T_H_2 and T_H_17 cells in lung single-cell suspensions. *Ex vivo* stimulation of these suspensions with a CXCL1/2/3/5 cocktail induced CXCR1/2-dependent proliferation of IL-4^+^, IL-5^+^, IL-13^+^, and IL-17^+^ T cells. *In vivo,* allergen challenge increased CXCR1/2-dependent accumulation of CXCR1^+^ and CXCR2^+^ T_H_2 and T_H_17 cells in the lungs, upregulated *Il6* and *Il23* expression in granulocytes/cytokines that support T_H_17 responses, and exacerbated eosinophilic lung inflammation. Concurrent CXCR1 and CXCR2 blockade effectively abrogated or attenuated these effects on dual T_H_2/T_H_17 allergic inflammation.

**Conclusions:**

CXCL chemokines play a novel role in driving proliferation of CXCR1^+^/CXCR2^+^ T_H_2/T_H_17 cells, thereby promoting overlapping T_H_2/T_H_17 lung inflammation. Targeting the CXCL-CXCR1/2 axis may provide a new strategy for treating dual T_H_2/T_H_17 diseases.

Asthma is a chronic inflammatory disease of the airways characterized by persistent eosinophilic and neutrophilic allergic inflammation. CD4^+^ T_H_2 cells producing IL-4, IL-5, and IL-13 and T_H_17 cells producing IL-17 are central drivers of this inflammation.^1^ IL-4 stimulates T_H_2 differentiation of CD4^+^ T cells by upregulating its master regulator, GATA3,^2^ and these T_H_2 cells drive allergic lung inflammation.^2^^,^^3^ IL-23, IL-1β, and IL-6 stimulate CD4^+^ T cells to differentiate toward T_H_17 through induction of their lineage-defining transcription factor retinoic acid–related orphan receptor gamma t (RORγt),^4^, ^5^, ^6^, ^7^ and these cells contribute to neutrophil recruitment.^1^

Chemokine receptors of the CC family are expressed on T_H_2 and T_H_17 cells,^8^, ^9^, ^10^, ^11^, ^12^ and they modulate eosinophilic and neutrophilic airway inflammation.^13^ Unlike the plethora of studies linking chemokine receptors of the CC family with allergic inflammation, the role of CXC-chemokine receptors (CXCRs) in modulating T_H_2 and T_H_17 cell–mediated responses remains poorly defined. This is surprising, given that C-X-C motif chemokine ligand (CXCL) chemokines—ligands for CXCR1/2—are elevated in the asthmatic airway.^14^^,^^15^ We and others have reported increased CXCL1, CXCL5, and CXCL8 levels in bronchoalveolar lavage fluid (BALF) and endobronchial biopsy samples from asthmatic subjects,^14^^,^^15^ particularly those with severe disease.^14^

CXCR1 and CXCR2 are G protein-coupled chemokine receptors that bind CXCL1, CXCL2, CXCL3, CXCL5, CXCL6, CXCL7, and CXCL8; they are expressed on various immune and lung structural cells.^16^^,^^17^ We previously reported that the CXCR2 small-molecule inhibitor SB225002 and dual CXCR1/2 inhibitor reparixin suppress allergic inflammation and serum IgE levels in cat dander extract (CDE)-sensitized and challenged mice.^18^^,^^19^ More recently, the CXCR1/2 inhibitor ladarixin has also been shown to reduce both eosinophilic and neutrophilic inflammation.^20^ However, that study did not directly assess the ability of ladarixin to block recruitment and proliferation of T_H_2 and T_H_17 cells. In the present study, we address this gap by performing flow cytometry (FCM) to elucidate the role of CXCL-CXCR1/2 axis in allergen and CXCL chemokine-induced recruitment and proliferation of T_H_2 and T_H_17 cells.

## Methods

### Allergenic extracts

Lyophilized CDE (lot nos. 253320, 351876, 392583) was obtained from Greer Laboratories (Lenoir, NC). The endotoxin level in CDE was measured with a LAL chromogenic endotoxin quantitation kit (Thermo Scientific, Hudson, NH) and was less than 0.1 pg/μg CDE protein, and thus unlikely to contribute significantly to inflammation.^21^

### Protocols used for animal studies

C57BL/6 mice were anesthetized with an intraperitoneal injection of a low dose of a xylazine/ketamine anesthetic mixture for intranasal administration of CDE and humanely killed by a lethal dose of the same anesthetic. All animal procedures were approved by the Institutional Animal Care and Use Committee of Baylor College of Medicine.

### CDE multiple challenge model

We reported that the CDE multiple challenge model (MCM) induces allergic sensitization and allergic lung inflammation associated with eosinophil recruitment and periodic acid–Schiff–positive mucin production in epithelial cells and elevated allergen-specific IgE levels in serum.^18^^,^^22^^,^^23^ Briefly, naive wild-type mice (male and female, 8-12 weeks old, 20-30 g) were sensitized by 5 intranasal challenges of CDE (100 μg/60 μL) on days 0, 1, 2, 3, and 4. After a rest period of 7 days, these mice were challenged with an intranasal dose of CDE or phosphate-buffered saline (PBS) on day 11 and humanely killed at 2, 4, 16, 28, 40, and 72 hours after CDE challenge.^18^^,^^22^^,^^23^ Some mice challenged with CDE on day 11 were orally treated with 15 mg/kg body weight of ladarixin or vehicle control on days 11, 12, and 13.

**Fig 1:**
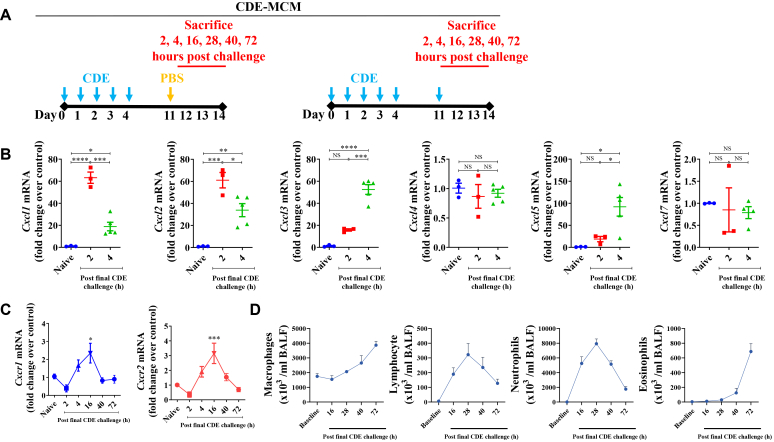
Allergen challenge upregulates lung CXCLs and recruits CXCR1/2-expressing T_H_2 cells and T_H_17 cells *in vivo,* and chemokines stimulate proliferation of T_H_2 cells and T_H_17 cells *ex vivo*. **(A)** Experimental protocols for CDE-MCM (with or without ladarixin treatment). **(B)***Cxcl* expressions in lungs at 2 and 4 hours after CDE challenge in CDE-MCM. **(C)** Time kinetics of *Cxcr1/2* expression in bone marrow cells in CDE-MCM. **(D)** Time course of BALF cell infiltration after CDE challenge in CDE-MCM.

### CDE single challenge model

The CDE single challenge model (SCM) induced innate lung inflammation without sensitization. Nonsensitized naive wild-type mice were intranasally challenged with a single dose of 100 μg/60 μL of CDE and humanely killed 28 hours after CDE challenge.

### Ladarixin

Good Manufacturing Practice human use–grade ladarixin was used in all the studies we performed and was kindly provided by the Dompe pharmaceutical company (Dompé farmaceutici, L’Aquila, Italy).

### Processing of mouse BALF and lung tissue samples

The BALF and lung samples were obtained as described previously.^22^

### Isolation of bone marrow cells

Bone marrow cells were isolated from the femur and tibia bones of the mouse. After red blood cell lysis by red blood cell lysing buffer (Sigma-Aldrich, St Louis, Mo), the bone marrow cells were frozen for subsequent experiments.

### Quantitative real-time PCR analysis

Total RNA from mouse lungs, bone marrow cells, or BALF cells were extracted with an RNeasy kit (Qiagen, Valencia, Calif). cDNA was synthesized with a cDNA Synthesis kit (Qiagen). Amplification by real-time PCR was performed on a CFX Connect Real-Time PCR Detection System (Bio-Rad, Hercules, Calif) with SYBR Green PCR Master Mix Kit (Bio-Rad) to examine lung mRNA expression of *Cxcl1, Cxcl2, Cxcl3, Cxcl4, Cxcl5, Cxcl*7, *Cxcr1, Cxcr2*. *Gata3, Il1b, Il13, Il17, Il4, Il5, Il6, Rorc,* and *Tgfb1.* These primers were purchased from Integrated DNA Technologies (Coralville, Iowa).

### Isolation of lung single cells

Lung tissues from mice were cut into small pieces and incubated with liberase–thermolysin medium (Sigma-Aldrich) in 42 μg/mL and 20% heat-inactivated fetal bovine serum in Hanks balanced salt solution at 37°C for 20 minutes. After passing the digested lung through a 70 μm cell strainer, ice-cold PBS was added to neutralize the enzyme activity. After several wash steps with PBS, the lung single cells were used for FCM analysis or were cultured.

### FCM analysis with lung single cells

Lung single cells were preincubated with TruStain FcX solution (BioLegend, San Diego, Calif) for 10 minutes at 4°C, then stained with fluorophore-conjugated antibodies Fixable Viability Dye eFluor 780 (eBioscience, San Diego, Calif), CD45 monoclonal antibody-pacific orange (Thermo Fisher Scientific, Waltham, Mass, catalog MCD4530, clone 30-F11), anti-mouse CD3–Alexa Fluor 700 (BioLegend, 100216, clone 17A2), rat anti-mouse CD4–Brilliant Violet 520 (BD Biosciences, San Jose, Calif; 563106, clone RM4.5), PE rat anti-mouse CD181 (CXCR1) (BD Biosciences, 566383, clone U45-632), BV711 rat anti-mouse CD182 (CXCR2) (BD Biosciences, 747812, clone V48-2310), BV786 rat anti-mouse SiglecF (BD Biosciences, 740956, clone E50-2440), Spark YG 593 anti-mouse Ly-6G antibody (BioLegend, 127668, clone 1A8), and Spark NIR 685 anti-mouse/human CD11b antibody (BioLegend, 101278, clone M1/17) at 1:100 dilution in flow stain buffer containing fetal bovine serum for 30 minutes at 4°C. Then cells were permeabilized with a Fixation/Permeabilization kit (BD Biosciences) according to the manufacturer’s instructions and stained with intracellular cytokine-specific antibodies: rat anti-mouse Rorγt^−^ Brilliant Violet-650 (BD Biosciences, 564722, clone Q31-378), mouse anti-GATA3 Alexa Fluor F488 (BD Biosciences, 560163, clone L50-823), rat anti-mouse IL-4–PE/Cyanine7 (BD Biosciences, 560699, clone 11B11), anti-mouse/anti-human IL-5–allophycocyanin (BD Biosciences, 554396, clone TRFK5), IL-13 monoclonal antibody (eBio13A), Brilliant Ultra Violet 805 (eBioscience), and rat anti-mouse IL-17A–PE (BD Biosciences, 559502, clone TC11-18H10) for 45 minutes at 4°C. After washing, FCM was performed with a high-parameter Cytek Aurora flow cytometer (Cytek Biosciences, Fremont, Calif). Staining specificity was determined by fluorescence-minus-one (FMO) control to enhance the reliability of the gating analysis. Absolute cell numbers were quantified by Precision Count Beads (BioLegend). The FCM data were analyzed by FlowJo 10.8.1 software (Becton Dickinson, Franklin Lakes, NJ). To enhance clarity of the gating analysis, representative FCM plots showing the gating strategies and corresponding FMO controls are provided in Figs E1-E3 in the Online Repository available at www.jaci-global.org. Neutrophils were identified as live CD45^+^CD11b^+^Ly6G^+^SiglecF^−^ cells. Eosinophils were identified as live CD45^+^CD11b^+^SiglecF^+^Ly6G^−^ cells. CD4^+^ T cells were identified as live CD45^+^CD3^+^CD4^+^ cells.

### Measurement of serum total IgE and CDE-specific IgE

The methods for measuring IgE have been described previously.^22^ Briefly, plates were coated with CDE overnight or with rat anti-mouse IgE (BD Biosciences) for 2 hours. After blocking with Sea Block buffer for 2 hours, serum from the mice was added. After washing, biotin-conjugated rat anti-mouse IgE (BD Biosciences) was added and incubated with avidin-conjugated alkaline phosphatase for 45 minutes at 4°C (Sigma-Aldrich). Fluorescence intensities were measured with AttoPhos Substrate Solution (Promega, Madison, Wis) by the Varioskan LUX reader (Thermo Fisher Scientific).

### Culture of lung single cells with chemokine cocktail

Lung single-cell suspensions (4 × 10^5^ cells) from sensitized mice 3 days after challenge were cultured in RPMI 1640 medium supplemented with l-glutamine, 2-mercaptoethanol, 10% fetal bovine serum, and 1% penicillin–streptomycin in 24-well plates, with or without a chemokine cocktail (CXCL1, CXCL2, CXCL3, and CXCL6, each at 250 ng/mL),^24^, ^25^, ^26^ for 4 days at 37°C. Where indicated, cells were pretreated with 10 μmol ladarixin for 1 hour before chemokine stimulation. On day 4, after washing the cells, the cells were preincubated with brefeldin A for 1 hour, then processed as described above. On day 5, the cells were applied for FCM.

### Statistical analysis

Statistical analysis was performed by unpaired *t* test for comparison of 2 groups or ANOVA for 3 or more groups by GraphPad Prism 6 (GraphPad Software, San Diego, Calif). The results are shown as means ± SEMs. All statistical analyses indicated data as significant at *P* < .05, with ∗*P* < .05, ∗∗*P* < .01, ∗∗∗*P* < .001, and ∗∗∗∗*P* < .0001.

## Results

### CDE challenge increases *Cxcl* mRNA expression in lung cells and CXCR1/2 in bone marrow cells of sensitized mice

Mice were sensitized to CDE by subjecting them to the CDE-MCM protocol (Fig 1, *A*).^19^^,^^22^ Compared to PBS challenge, CDE challenge induced significantly increased lung expression of *Cxcl1, Cxcl2, Cxcl3,* and *Cxcl5* at 2 hours and/or 4 hours (Fig 1, *A*), but not *Cxcl4* or *Cxcl7* (Fig 1, *B*). A shared feature of these upregulated CXCL chemokines is that they bind to CXCR1/2.^27^^,^^28^ We reasoned that expression and secretion of these CXCL chemokines in the lungs should increase the number of progenitor cells expressing CXCR1/2 in the bone marrow, preparing them for migration to the lungs. To test this hypothesis, we examined the kinetics of CDE challenge–induced upregulation of *Cxcr1/2* mRNA expression in bone marrow of sensitized mice. CDE challenge increased the mRNA expression of *Cxcr1/2* bone marrow cells at 16 hours after CDE challenge (Fig 1, *C*), suggesting that these receptors play a role in recruiting inflammatory cells from bone marrow to the lungs.

To determine the kinetics of inflammatory cell infiltration after CDE exposure, we analyzed differential cell counts in the BALF after CDE challenge in the CDE-MCM model (Fig 1, *A*). CDE challenge induced a rapid and transient increase in neutrophils and lymphocytes, which peaked at 28 hours (Fig 1, *E*). Eosinophil numbers began to rise later, becoming evident at 72 hours after challenge (Fig 1, *E*). Macrophage numbers showed a more gradual increase, which peaked at 72 hours (Fig 1, *E*).

### CDE challenges increases CXCR1/2-positive T cells in the lung

**Fig 2:**
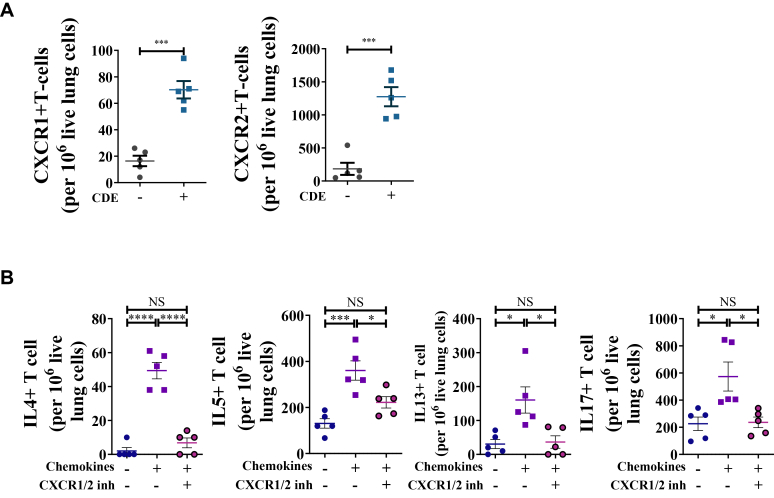
CXCR1/2 expression on lung T cells and their response to CXCL chemokines. **(A)** Numbers of CXCR1/2-expressing T cells in lung tissue. **(B)** Numbers of IL-4–, IL-5–, IL-13–, and IL-17–producing T cells in lung cells cultured with cocktail of CXCL chemokines. Data are representative of 3 independent experiments (n = 3-5 per group). Individual data points are shown.

FCM revealed that splenic naive CD4^+^ T-cell expression levels of CXCR1/2 was similar to the corresponding FMOs (Fig E2). Given that CDE challenge in the CDE-MCM model (Fig 1, *A*) upregulated *Cxcl1, Cxcl2, Cxcl3,* and *Cxcl5* (Fig 1, *B*), we hypothesized that CD4^+^ T cells recruited to the lung after CDE challenge express CXCR1 or CXCR2. Consistent with our hypothesis, FCM revealed that CD4^+^ T cells in the lung tissue of CDE-challenged mice expressed CXCR1 and/or CXCR2 (Fig 2, *A*), indicating that these receptors are likely induced on allergen challenge or allergic sensitization. On day 11 before allergen challenge, neutrophils were virtually undetectable in lung cell suspensions (data not shown), ruling out significant effects from residual neutrophils.

### CXCL-CXCR1/2 axis stimulates proliferation of lung T_H_2 and T_H_17 cells

On the basis of these findings, we hypothesized that CXCL-CXCR1/2 signaling promotes the proliferation of CXCR1^+^ and CXCR2^+^ T_H_2 cells and T_H_17 cells in the lungs. To test this, lung single-cell suspensions from CDE-MCM mice were stimulated with a chemokine cocktail consisting of CXCL1, CXCL2, CXCL3, and CXCL5 in the presence or absence of ladarixin, then subsequently analyzed by FCM. Stimulation with CXCL chemokines increased the number of T_H_2 cells expressing IL-4, IL-5, and IL-13, as well as T_H_17 cells expressing IL-17 (Fig 2, *B*), suggesting that CXCR1/2 signaling can contribute to the expansion of these T-cell subsets in mixed lung cell cultures. To elucidate the role of the CXCL-CXCR1/2 axis in stimulating CXCL-mediated expansion of T_H_2 and T_H_17 cells, we used an allosteric inhibitor of both CXCR1 and CXCR2 with ladarixin, a small molecule that binds to the allosteric pocket in the transmembrane region of both receptors with over 100-fold greater affinity than first-generation CXCR1/2 inhibitors.^29^ CXCL-mediated proliferation of T_H_2 and T_H_17 cells was partially inhibited by ladarixin (Fig 2, *B*), consistent with a role for the CXCL-CXCR1/2 axis in promoting T-cell expansion in these mixed lung cell cultures.

### Dual inhibition of CXCR1 and CXCR2 blocks allergen challenge-induced T_H_2- and T_H_17-associated mRNA expression

**Fig 3:**
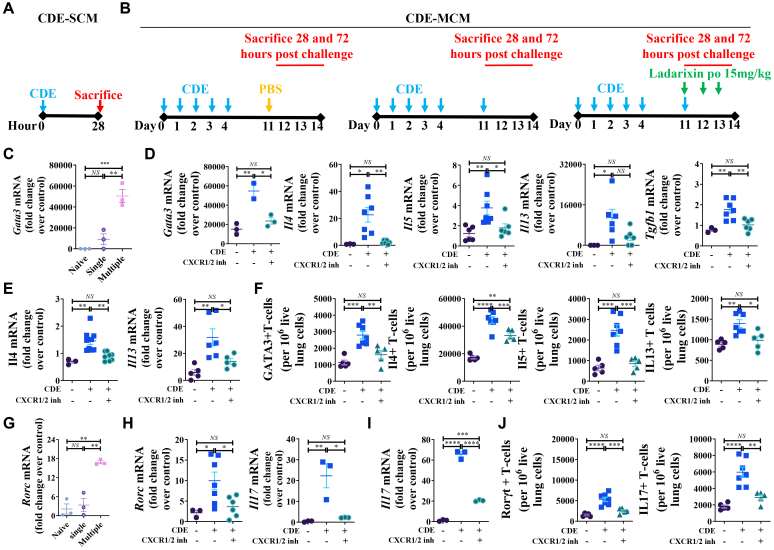
Concurrent inhibition of CXCR1 and CXCR2 blocks T_H_2- and T_H_17-associated gene expression in CDE-challenged lungs. **(A)** Experimental protocols for CDE-SCM. **(B)** Experimental protocols for CDE-MCM (with or without ladarixin treatment). **(C)***Gata3* mRNA expressions in BALF cells after CDE challenge in CDE-SCM and CDE-MCM mice at 28 hours after challenge. **(D-F)** At 28 hours after CDE challenge in CDE-MCM. *(D) Gata3, Il4, Il5, Il13,* and *Tgfb1* cells mRNA expressions in BALF cells at 28 hours after CDE challenge. *(E)* Lung mRNA expressions of *Il4* and *Il13* at 28 hours after CDE challenge. *(F)* Numbers of GATA3–, IL-4–, IL-5–, and IL-13–positive lung T_H_2 cells at 72 hours after CDE challenge. **(G)***Rorc* mRNA expressions in BALF cells after CDE challenge in CDE-SCM and CDE-MCM mice at 28 hours after CDE challenge. **(H)***Il17* and *Rorc* mRNA expressions in BALF cells in CDE-MCM at 28 hours after CDE challenge. **(I)***Il17* mRNA expressions in lung in CDE-MCM at 28 hours after CDE challenge. **(J)** Numbers of RORγt-positive T cells and IL-17–positive T_H_17 cells in lung in CDE-MCM at 72 hours after CDE challenge. Data are representative of 3 independent experiments (n = 3-7 per group). Individual data points are shown.

Given these findings, we next investigated whether blockade of CXCR1/2 signaling could attenuate T_H_2- and T_H_17-mediated gene expression in allergen-challenged lungs. GATA3, a master transcription factor of T_H_2 differentiation, is central to allergic lung inflammation.^2^^,^^3^ We examined *Gata3* mRNA expression in BALF cells from naive mice as well as mice subjected to CDE-SCM (Fig 3, *A*) and CDE-MCM protocols (Fig 3, *B*). Compared to naive mice, CDE challenge in the CDE-SCM protocol failed to increase *Gata3* expression in BALF cells (Fig 3, *C*), whereas CDE challenge in CDE-MCM protocol upregulated *Gata3* in BALF cells relative to both naive mice and CDE-SCM mice (Fig 3, *C*). In addition, CDE-MCM mice exhibited increased *Il4, Il5, Il13,* and *Tgfb1* in BALF cells (Fig 3, *D*), *Il4* and *Il13* in lung tissues (Fig 3, *E*), and enhanced recruitment of GATA3^+^, IL-4^+^, IL-5^+^, and IL-13^+^ T_H_2 cells to the lungs (Fig 3, *F*). Notably, all of these responses were abrogated by ladarixin treatment (Fig 3, *D-F*). We further investigated whether CDE challenge induces T_H_17-associated gene expression in the lungs. CDE challenge in the CDE-SCM model failed to increase *Rorc* expression in BALF cells (Fig 3, *G*), whereas the CDE-MCM protocol significantly upregulated *Rorc* mRNA expression relative to both naive and CDE-SCM groups (Fig 3, *G*). CDE challenge in CDE-MCM also upregulated *Il17* mRNA expression in BALF cells (Fig 3, *H*), elevated *Il17* mRNA in lung tissues (Fig 3, *I*), and increased the number of RORγt^+^ and IL-17^+^CD4^+^ T cells (Fig 3, *J*).

### Dual inhibition of CXCR1 and CXCR2 blocks allergen challenge-induced recruitment of T_H_2 cells and T_H_17 cells

**Fig 4:**
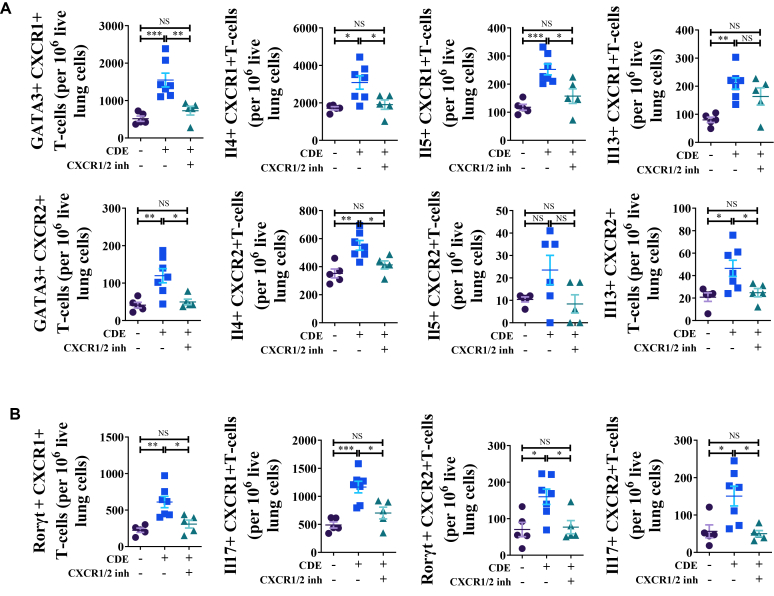
Concurrent inhibition of CXCR1 and CXCR2 blocks recruitment of T_H_2 cells and T_H_17 cells. **(A)** Numbers of CXCR1/2-expressing GATA3–, IL-4–, IL-5–, and IL-13–positive lung T_H_2 cells at 72 hours after CDE challenge in CDE-MCM. **(B)** Numbers of CXCR1/2-expressing RORγt- and IL-17–positive T_H_17 cells in lung at 72 hours after CDE challenge in CDE-MCM. Data are representative of 3 independent experiments (n = 5-7 per group). Individual data points are shown.

Next, we hypothesized that T_H_2 and T_H_17 cells themselves express CXCR1/2 receptors. To test this, we quantified the number of CXCR1^+^ and CXCR2^+^ T_H_2 cells and T_H_17 cells in the lungs of CDE-MCM mice. CDE challenge significantly increased the recruitment of CXCR1^+^ and CXCR2^+^ CD4^+^ T cells coexpressing GATA3, IL-4, IL-5, and IL-13 (Fig 4, *A*), as well as RORγt and IL-17 into the lungs (Fig 4, *B*). Ladarixin treatment (Fig 1, *A*) abrogated or vigorously blocked recruitment of these cells, with the exception of CXCR1^+^IL-13^+^CD4^+^ T cells, suggesting selective sensitivity to receptor inhibition.

### Dual inhibition of CXCR1 and CXCR2 blocks allergen challenge-induced IL-23 expression and secretion

**Fig 5:**
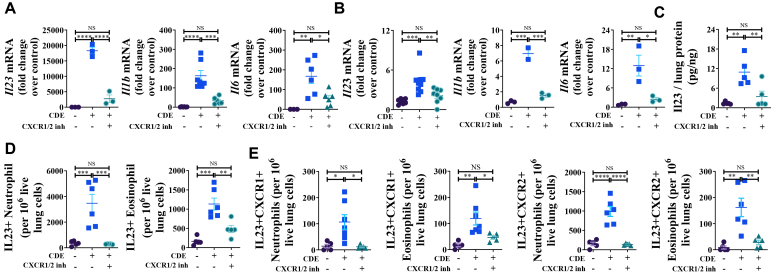
CXCR1/2 upregulate T_H_17-stimulating cytokine mRNA and protein expression. (**A** and **B**) *Il23, Il1b,* and *Il6* mRNA expressions in *(A)* BALF cells and *(B)* lung in CDE-MCM at 28 hours after CDE challenge. **(C)** Protein levels of *Il23* in lung in CDE-MCM at 28 hours after CDE challenge. **(D)** Numbers of IL-23–positive neutrophils and eosinophils in lung in CDE-MCM at 72 hours after CDE challenge. **(E)** Numbers of CXCR1- and CXCR2-expressing IL-23–positive neutrophils and eosinophils in lung in CDE-MCM at 72 hours after CDE challenge. Data are representative of 3 independent experiments (n = 3-7 per group). Individual data points are shown.

IL-23 is a key cytokine that promotes the survival of T_H_17 cells and mediates T_H_17 inflammation.^30^ Prior studies have shown that IL-23, IL-1β, and IL-6 are elevated in the BALF obtained from the subjects with asthma^31^^,^^32^ and that these cytokines drive T_H_17 differentiation^4^, ^5^, ^6^ by upregulating its master regulator RORγt.^7^ Building on our observation that dual inhibition of CXCR1/2 suppresses T_H_17-associated cytokine expression and cell recruitment, we hypothesized that stimulation of CXCR1/2 after allergen challenge contributes to upregulation of *Il23, Il1b,* and *Il6*. Consistent with our hypothesis, CDE challenge in CDE-MCM mice (Fig 1, *A*) upregulated *Il23, Il1b,* and *Il6* mRNA expression in BALF cells (Fig 5, *A*) and lung tissues (Fig 5, *B*). This was accompanied by an increase in IL-23 protein levels in lung tissues (Fig 5, *C*) as well as increased numbers of IL-23^+^ neutrophils and eosinophils (Fig 5, *D*), including CXCR1^+^ and CXCR2^+^ IL-23^+^ subsets (Fig 5, *E*). All these effects were abrogated by ladarixin treatment (Fig 1, *A,* and Fig 5). In addition to granulocytes, we also evaluated whether other lung-resident or infiltrating cell populations contribute to IL-23 production after CDE challenge. Dendritic cells, macrophages, T cells, endothelial cells, and epithelial cells exhibited detectable IL-23 expression after CDE challenge (see Fig E4 in the Online Repository available at www.jaci-global.org). Importantly, ladarixin treatment significantly reduced IL-23 production across all examined cell types, with the exception of endothelial cells. These findings indicate that CXCR1/2 inhibition attenuates allergen-induced IL-23 induction across multiple structural and immune cell compartments, not only within granulocytes.

### Dual inhibition of CXCR1 and CXCR2 attenuates allergen challenge-induced allergic lung inflammation

**Fig 6:**
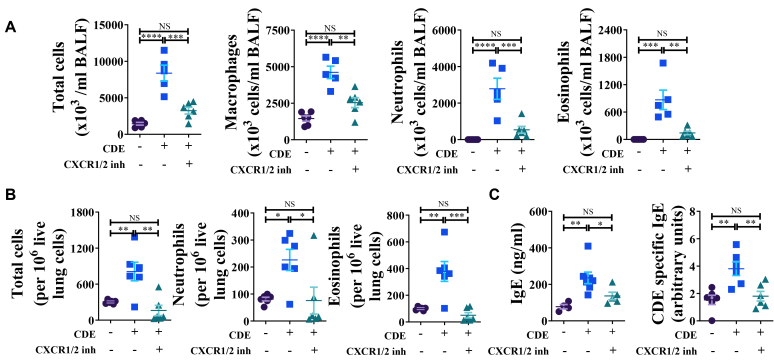
CXCR1/2 stimulate allergic lung inflammation and allergen-specific IgE. **(A)** Numbers of inflammatory cells in BALF at 72 hours after CDE challenge in CDE-MCM. **(B)** FCM analysis of numbers of inflammatory immune cells in lungs at 72 hours after CDE challenge in CDE-MCM. **(C)** Levels of serum total IgE and CDE-specific IgE at 72 hours after CDE challenge in CDE-MCM. Data are representative of 3 independent experiments (n = 4-7 per group). Individual data points are shown.

Next, we examined whether dual inhibition of CXCR1/2 blocks allergic airway inflammation. Compared to PBS-challenged mice in the CDE-MCM protocol (Fig 1, *A*), CDE challenge increased the numbers of total cells, macrophages, neutrophils, and eosinophils in BALF (Fig 6, *A*) and lung tissues, as quantified by FCM (Fig 6, *B*) at 72 hours after the final CDE challenge, as well as levels of total IgE and CDE-specific IgE in serum (Fig 6, *C*). Treatment with ladarixin in the CDE-MCM model (Fig 1, *A*) reduced the numbers of total cells and macrophages in the BALF (Fig 6, *A*), abrogated the increase in neutrophil and eosinophil numbers into the BALF (Fig 6, *A*) and lung compartments (Fig 6, *B*), and decreased levels of total and CDE-specific IgE in serum (Fig 6, *C*).

## Discussion

To our knowledge, this is the first study to demonstrate that concurrent blockade of CXCR1/2 effectively blocks dual T_H_2/T_H_17-mediated inflammation. Our prior work implicated neutrophilic inflammation and IL8/CXCR2 signaling in severe asthma.^15^ The present study extends these findings by demonstrating that CXCL-CXCR1/2 signaling directly promotes the accumulation of CXCR1/2^+^ T_H_2 and T_H_17 cells in the lungs, leading to dual T_H_2/T_H_17 inflammation.

Although several CXCR family members are associated with specific CD4^+^ T-cell subsets, the functional role of CXCR1/2 signaling in allergen-specific T_H_2 and T_H_17 responses has remained unclear. CXCR3-deficient mice exhibited reduced numbers of IFN-γ^+^ CD4^+^ T cells and a relative increase in IL-13^+^CD4^+^ T cells in the lungs.^33^ Human T_H_2 cells express CCR3 and CCR4, but not CXCR3.^34^ In contrast, human T_H_1 cells express high levels of CXCR3 and CCR5.^34^ There is a significantly higher frequency of CXCR1-expressing CD4^+^ T cells in allergic individuals than in healthy controls, suggesting a potential association between CXCR1^+^ T cells and allergic conditions.^35^

Other chemokine receptors, such as CXCR3-5, have been linked to specific T-cell subsets, underscoring the importance of chemokine signaling in T-cell differentiation.^36^, ^37^, ^38^ Our findings show that CXCL-CXCR1/2 signaling amplifies allergic inflammation by promoting the expansion and cytokine production of T_H_2 and T_H_17 cells, suggesting this pathway as a potential therapeutic target.

**Fig 7:**
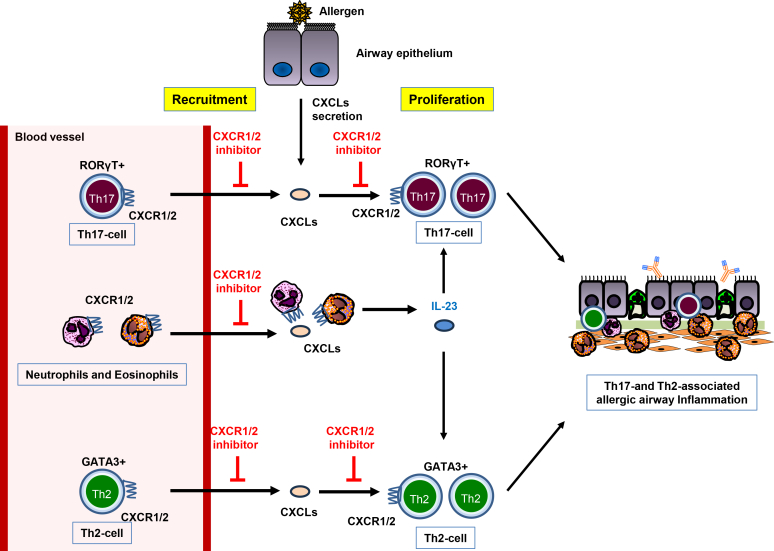
Schematic diagram showing CXCL-CXCR1/2 signaling stimulates T_H_2 and T_H_17 allergic lung inflammation. Allergen challenge induces secretion of CXCL chemokines that recruit IL-23–producing neutrophils and eosinophils to airways. IL-23 stimulates naive CD4^+^ T cells to differentiate into T_H_2 and T_H_17 cells. CXCL chemokines stimulate proliferation of T_H_2 cells and T_H_17 cells. Inhibition of CXCL-CXCR1/2 pathways suppresses both T_H_2- and T_H_17-associated allergic lung inflammation.

CD4^+^ T_H_2 and T_H_17 cells contribute to allergic airway inflammation through the secretion of T_H_2 and T_H_17 cytokines, which in turn promote the recruitment of eosinophils^2^^,^^3^ and neutrophils,^39^ respectively. Importantly, epithelial cells can rapidly produce chemokines such as eotaxin and CXCLs before T-cell recruitment, thereby amplifying T_H_2- and T_H_17-type responses. We previously reported that allergen challenge stimulates MD2-TLR4-MyD88–mediated secretion of CXCL chemokines.^18^^,^^22^^,^^23^ However, the contribution of the CXCL-CXCR axis to T_H_2- and T_H_17-associated allergic lung inflammation was not explored in those or other reports. Here, we show for the first time that dual inhibition of CXCR1 and CXCR2 abrogates the recruitment of T_H_2 cells as well as T_H_17 cells. In addition, dual inhibition suppressed the proliferation of both T_H_2 and T_H_17 cells. On the basis of these observations, we propose a model, shown in Fig 7, in which allergen challenge induces CXCL chemokine secretion in the airways, which in turn stimulates the proliferation of CXCR1^+^ and CXCR2^+^ T_H_2 and T_H_17 cells as well as secretion of T_H_2 and T_H_17 cytokines from these cells. Thus, simultaneous inhibition of CXCR1 and CXCR2 may represent an effective strategy to block dual T_H_2/T_H_17 pathology.

A limitation of our study is that the CXCL-mediated expansion of T_H_2 and T_H_17 cells was assessed in mixed lung cell suspensions. Therefore, we cannot exclude the possibility that other CXCR1/2-expressing lung cells may indirectly contribute to T-cell proliferation, and the direct effects of these chemokines on isolated CD4^+^ T cells remain to be determined.

In the present study, we show that dual inhibition of CXCR1 and CXCR2 signaling abrogates allergen challenge-induced IL-23 secretion. IL-23 plays a crucial role in maintaining T_H_17 cells^30^ and regulating T_H_2 and T_H_17 cells.^40^^,^^41^ IL-23 deficiency or blockage suppresses T_H_2 differentiation^40^ as well as IL-5 and IL-13 production and eosinophil recruitment.^41^ In patients with T_H_2/T_H_17-high severe asthma, elevated levels of IL-23 have been detected in BALF.^32^ Neutrophils and eosinophils can produce IL-23^42^^,^^43^ and may therefore amplify allergic inflammation. Our data suggest that CXCR1^+^ and CXCR2^+^ T_H_2 and T_H_17 cells are central players in allergic inflammation, and that modulation of IL-23 may be a downstream consequence of inhibiting granulocyte recruitment. In our model, CXCL chemokines activate CXCR1/2 on granulocytes and T cells, facilitating their recruitment and promoting IL-23 secretion. IL-23 in turn promotes T_H_2 and T_H_17 cytokine production. Dual inhibition of CXCR1 and CXCR2 may thus provide a unique solution to suppress the recruitment of IL-23–producing inflammatory neutrophils and eosinophils and directly limit T_H_2 and T_H_17 cell–driven allergic inflammation.

We previously reported that CXCR1/2 inhibitors mitigate recruitment of neutrophils in allergic inflammation,^18^^,^^19^ and the current study confirms that ladarixin suppresses total lung neutrophils. These data build on our earlier work demonstrating that reactive oxygen species–generating neutrophils exacerbate allergic airway inflammation and sensitization.^18^ Other studies have also supported an important role of neutrophils in local allergic responses, including atopic dermatitis,^44^ allergic contact dermatitis,^45^ anaphylaxis,^46^ and asthma,^18^^,^^23^ by regulating innate and adaptive immunity. Because neutrophils express abundant CXCR1/2,^47^ their inhibition by ladarixin likely contributes to the anti-inflammatory effects of ladarixin in our model.

Neutrophils express both CXCR1 and CXCR2, which have nonredundant roles in recruitment and activation. CXCL8 differentially activates these receptors,^48^ and selective inhibition of CXCR1—but not CXCR2—affects neutrophil function,^49^ thus highlighting the functional differences between the two receptors. SB225002, a highly selective CXCR2 antagonist (>150-fold over CXCR1), has been shown to reduce neutrophil recruitment in acute lung injury and other inflammatory models, focusing primarily on neutrophil-mediated processes rather than adaptive T helper cell responses.^50^ In contrast, reparixin, which inhibits CXCR1 and to some extent CXCR2, has shown efficacy in systemic fibrotic/inflammatory models, although its effects in allergen-induced T_H_2/T_H_17 airway inflammation remain poorly characterized.^51^^,^^52^ In this light, ladarixin’s dual blockade of CXCR1 and CXCR2 likely underlies the observed suppression of both T_H_2 and T_H_17 cell recruitment and proliferation in our model, suggesting that individual receptor antagonism may not fully recapitulate this effect.

A previous study by Mattos et al^20^ reported that dual CXCR1/2 inhibition by ladarixin improved neutrophil-dependent airway inflammation. In their short-term model, both T_H_2 and T_H_17 responses were effectively abrogated, whereas in a more chronic allergen exposure model, ladarixin treatment had only minor effects on T_H_2 inflammation.^20^ Consistent with the short-term observations, our current study using the CDE-MCM model demonstrates that dual CXCR1/2 inhibition suppresses the recruitment and proliferation of T_H_2 and T_H_17 cells. However, that study did not evaluate allergen and CXCL chemokine–induced recruitment and proliferation of T_H_2 and T_H_17 cells. In the present study, we addressed this gap by performing FCM to elucidate recruitment and proliferation of T_H_2 and T_H_17 cells and expansion of CXCR1/2^+^ T_H_2 and T_H_17 cells. Our data provide a cellular mechanism linking CXCL-CXCR1/2 signaling to dual T_H_2/T_H_17 inflammation. We acknowledge that, similar to Mattos et al, the effects on type 2 inflammation may be less pronounced in chronic or repeated allergen exposure models, and future studies are needed to assess the efficacy of ladarixin in such chronic settings.

Our observations identify a previously unrecognized role of CXCL chemokines in directly stimulating the proliferation of CXCR1^+^ and CXCR2^+^ T_H_2 and T_H_17 cells, thereby stimulating overlapping T_H_2/T_H_17 allergic lung inflammation. Targeting the CXCL-CXCR1/2 axis offers a novel strategy for simultaneously inhibiting T_H_2/T_H_17-driven allergic diseases.

**Clinical implication**

Allosteric inhibition of CXCR1/2 by administration of ladarixin should be investigated as a novel therapeutic strategy of mitigating T_H_2/T_H_17-associated allergic lung inflammation in humans.

## Disclosure statement

Supported in part by the 10.13039/100000005US Department of Defense (grant PR171425 W81XWH-18-1-0743), the 10.13039/100000050National Heart, Lung, and Blood Institute (grants 5R01HL145477-02 and 3R01HL145477-01S1) to S.S., and Nancy Chang Chair, Biology of Inflammation Center, Baylor College of Medicine to S.S.

Disclosure of potential conflict of interest: The authors declare that they have no relevant conflicts of interest.

## Supplementary data

Supplementary Fig E1

Supplementary Fig E2

Supplementary Fig E3

Supplementary Fig E4
