# Supplementary figures and images for: Targeting CXCR1/2 suppresses TH2/TH17 cell responses and inhibits dual-pathology allergic lung inflammation
Source: J Allergy Clin Immunol Glob. 2026 Apr 30;5(4):100726. doi: 10.1016/j.jacig.2026.100726 (PMC13218153; doi:10.1016/j.jacig.2026.100726)

Supplemental Figure 1

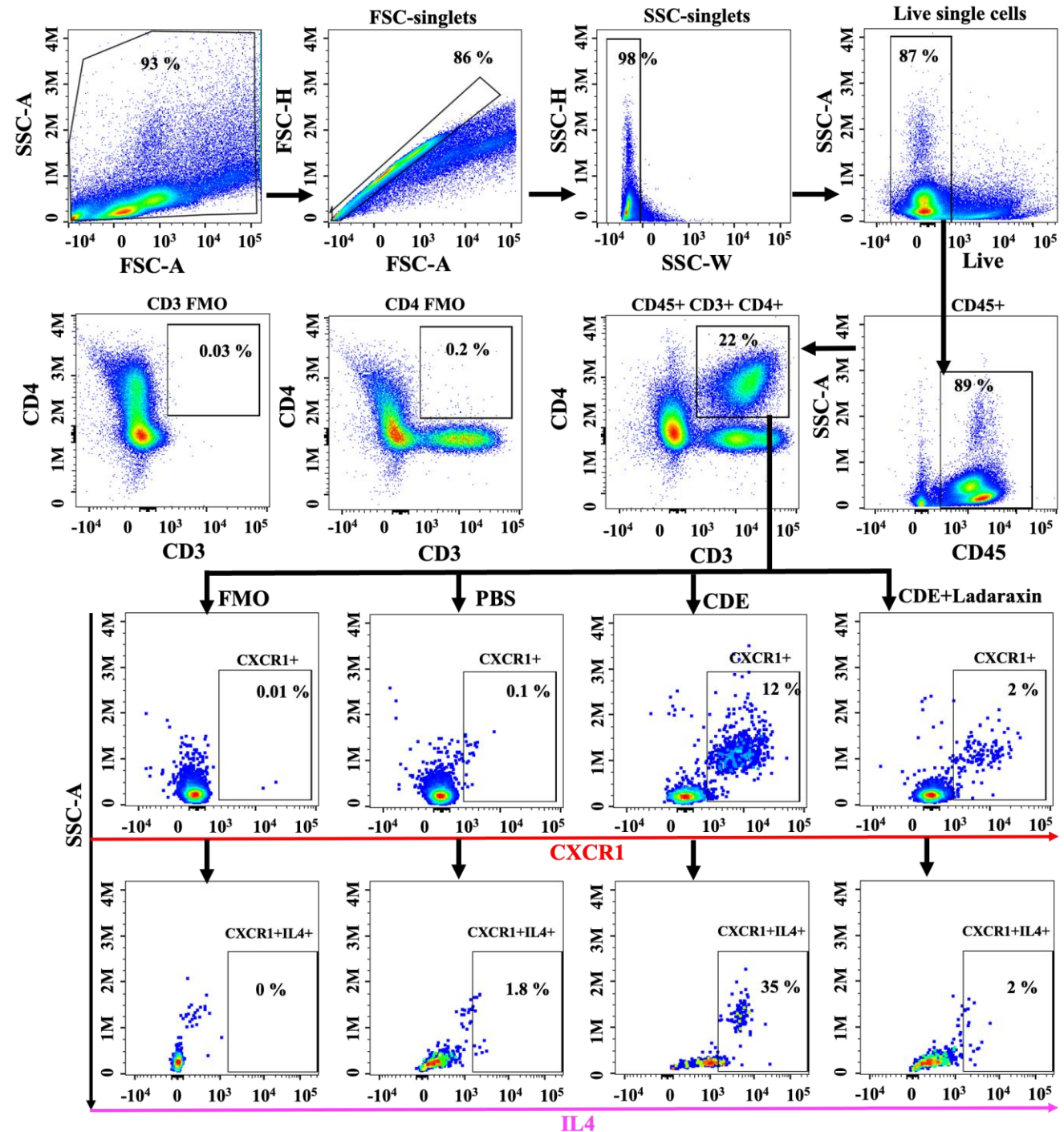

Supplement: Supplementary Fig E1 [file mmc1.pdf]

# Supplemental Figure 2

**A**

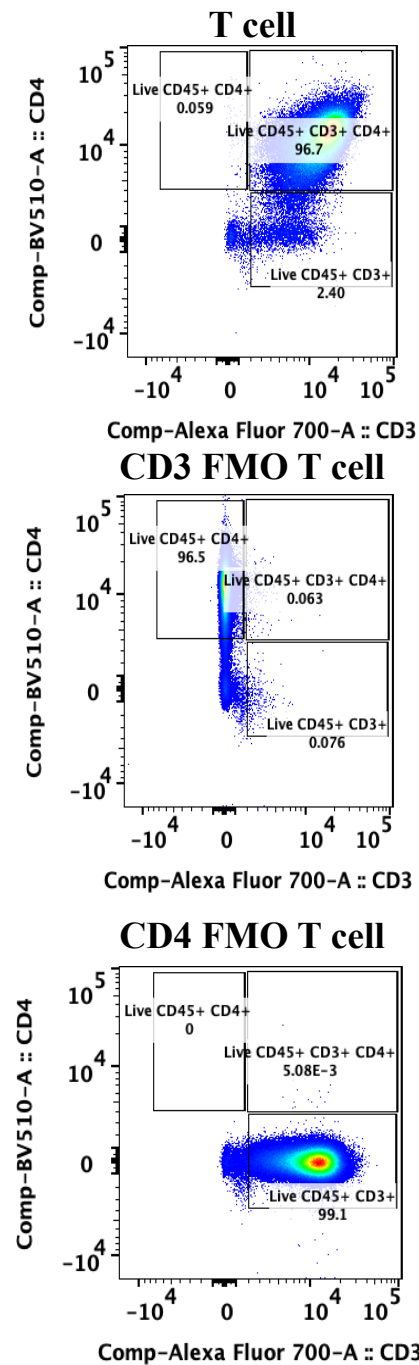

**B**

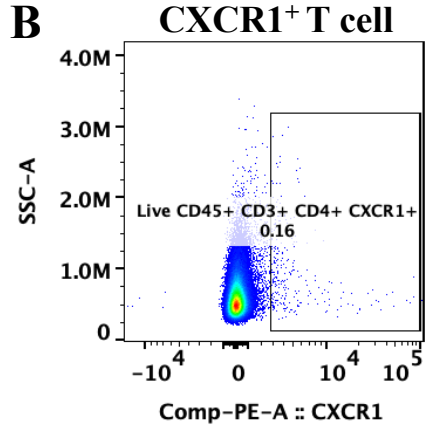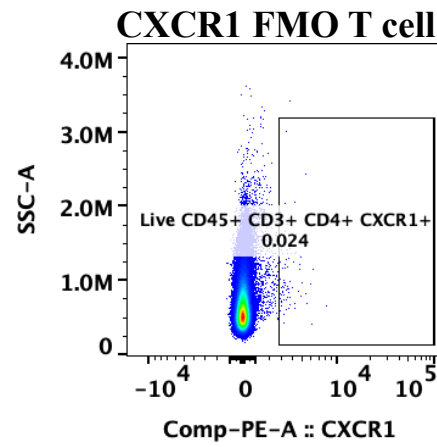

**C**

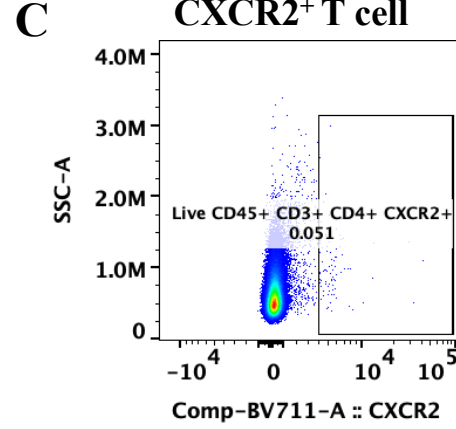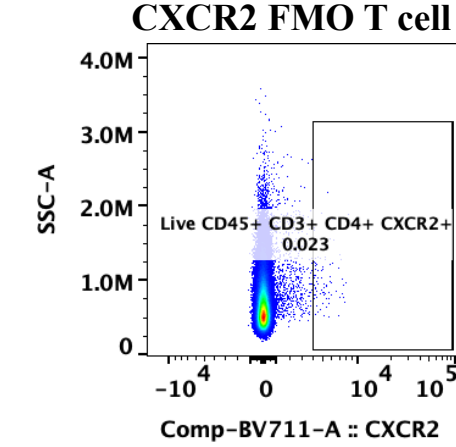

Supplement: Supplementary Fig E2 [file mmc2.pdf]

Supplemental Figure 3

A

CD45+CD3+CD4+IL17+FMO

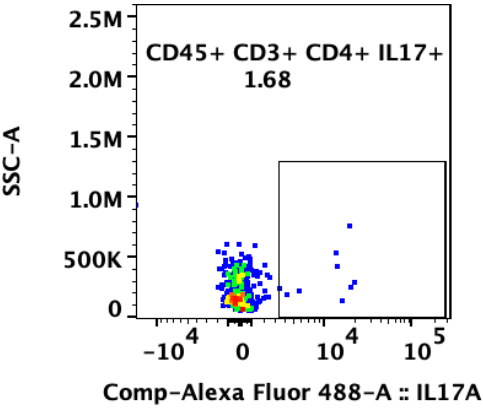

B

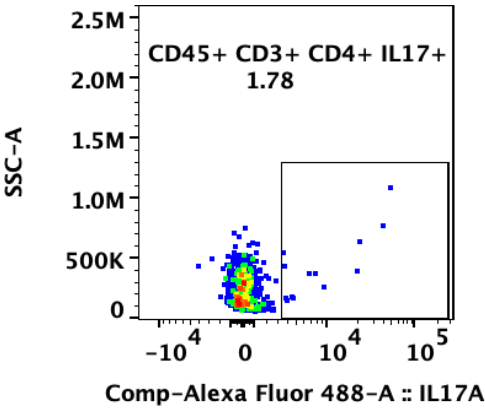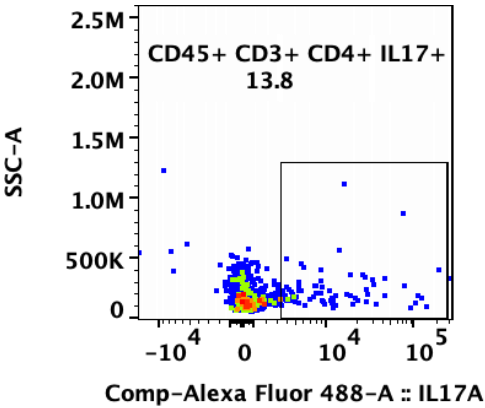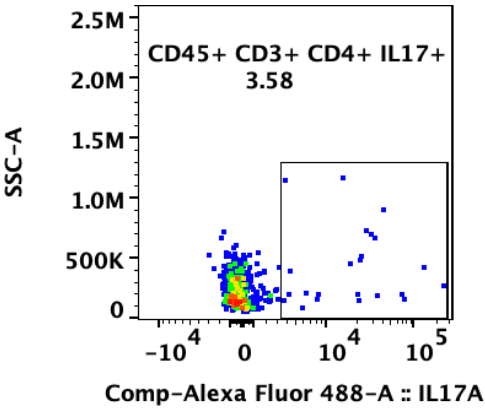

|             |   |   |   |
|-------------|---|---|---|
| Chemokines  | - | + | + |
| CXCR1/2 inh | - | - | + |

Supplement: Supplementary Fig E3 [file mmc3.pdf]

Supplemental Figure 4

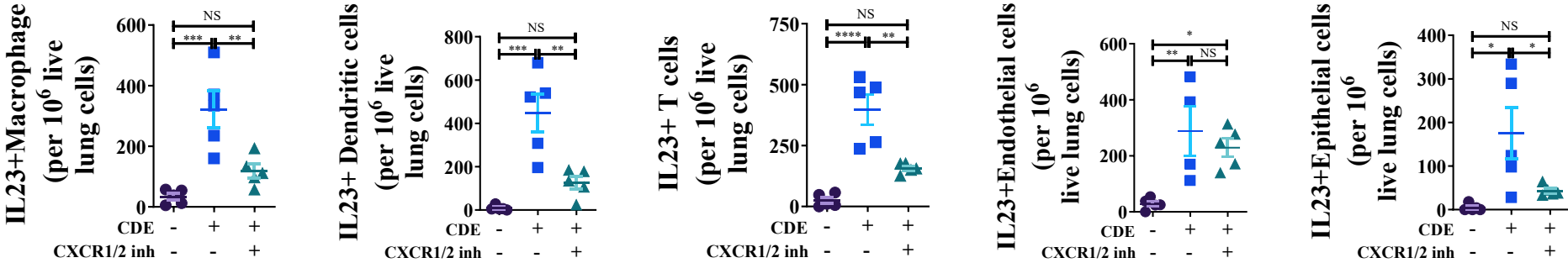

Supplement: Supplementary Fig E4 [file mmc4.pdf]
