# Supplementary material for: Targeting CXCR1/2 suppresses TH2/TH17 cell responses and inhibits dual-pathology allergic lung inflammation
Source: J Allergy Clin Immunol Glob. 2026 Apr 30;5(4):100726. doi: 10.1016/j.jacig.2026.100726 (PMC13218153; doi:10.1016/j.jacig.2026.100726)
Supplement: Supplementary Figs Legend [file mmc5.docx]

**Supplemental Figure Legend**

**Supplemental Figure 1. Representative FC plot gating strategy**

The forward and side scatter (FSC vs SSC) density plots were generated to separate cell populations of interest from debris. Next, doublets and debris were excluded by forward scatter height versus forward scatter area density plot (FSC-H vs FSC-A). Next, dead cells were excluded by gating only live cells using fluorophore-conjugated antibodies Fixable Viability Dye eFluor 780. In all subsequent steps, side scatter area (SSC-A) was plotted against fluorescence-tagged antibodies to gate positive cells by excluding cells in the corresponding FMO gate. Sequentially, live cells were gated using CD45+ cells to gate hematopoietic cells, CD3+, CD4+ double positive cells to gate for CD4+ T-cells, CXCR1+ cells to gate CXCR1-expressing CD4+ T-cells, then intracellular IL4+ to gate for CXCR1+ IL4+ Th2 cells in the lungs.

Abbreviations

FMO; fluorescence minus one

FSC; forward scatter

SSC; side scatter

**Supplemental Figure 2. Representative FC plots showing gating strategies and FMO controls for splenic naïve T cells.**

(**A**) Gating of naïve CD4⁺ T cells (Live CD45⁺CD3⁺CD4⁺), with the percentage displayed inside the gate (96.7%). The corresponding CD3 and CD4 FMO controls show gated fractions of 0.14% and 0.00%, respectively. (**B**) CXCR1 staining, with the percentage inside the CXCR1⁺ gate shown as 0.16%, compared with the CXCR1 FMO control (0.024%). (**C**) CXCR2 staining, with the percentage inside the CXCR2⁺ gate shown as 0.051%, compared with the CXCR2 FMO control (0.023%).

Abbreviations

FMO; fluorescence minus one

**Supplemental Figure 3. Representative FC plots showing gating strategies and FMO controls for cultured lung T cells.**Total CD45⁺CD3⁺CD4⁺ IL17⁺ T cells from lung cell cultures are shown, along with the corresponding CD45⁺CD3⁺CD4⁺ IL17⁺ FMO controls.

Abbreviations

FMO; fluorescence minus one

**Supplemental Figure 4. IL-23–producing lung structural and immune cells after CDE challenge**

The numbers of IL-23–positive dendritic cells, macrophages, T cells, endothelial cells, and epithelial cells in the lung in CDE-MCM at 72 h after CDE challenge.

Abbreviations

CDE; cat dander extract

CDE-MCM; CDE-multiple challenge model
